# Adult Onset Dysphagia: Right Sided Aortic Arch, Ductus Diverticulum, and Retroesophageal Ligamentum Arteriosum Comprising an Obstructing Vascular Ring

**DOI:** 10.1155/2017/9614835

**Published:** 2017-03-15

**Authors:** Ankur Sinha, Hitesh Raheja, Vinod Namana, Sunil Abrol, Stephan Kamholz, Vijay Shetty

**Affiliations:** ^1^Departments of Medicine and Cardiology, Maimonides Medical Center, Brooklyn, NY, USA; ^2^Department of Cardiothoracic Surgery, Maimonides Medical Center, Brooklyn, NY, USA; ^3^Divisions of Pulmonary Medicine and Critical Care Medicine, Maimonides Medical Center, Brooklyn, NY, USA

## Abstract

A 49-year-old African American male patient with no past medical history was admitted because of 3 months of difficulty swallowing solid and liquid foods. He had constant retrosternal discomfort and appeared malnourished. The chest radiograph revealed a right sided aortic arch with tracheal deviation to the left. A swallow study confirmed a fixed esophageal narrowing at the level of T6. Contrast enhanced Computed Tomography (CT) angiogram of the chest and neck revealed a mirror image right aortic arch with a left sided cardiac apex and a prominent ductus diverticulum (measuring 1.7 × 1.8 cm). This structure extended posterior to and indented the mid esophagus. A left posterolateral thoracotomy was performed and the ductus diverticulum was resected. A retroesophageal ligamentum arteriosum was found during surgery and divided. This rare combination of congenital anatomical aberrations led to severe dysphagia in our patient. Successful surgical correction in the form of resection of the ductus diverticulum and division of the retroesophageal ligamentum arteriosum led to complete resolution of our patient's symptoms.

## 1. Introduction

We present a patient with dysphagia due to a combination of congenital malformations. These included a right sided aortic arch, a prominent ductus diverticulum, and a retroesophageal ligamentum arteriosum. These malformations put together formed an obstructing vascular ring around the esophagus. We report this unusual combination of malformations with successful surgical correction leading to a favorable clinical outcome.

## 2. Presentation

A 49-year-old African American male patient with no past medical history was admitted because of 3 months of difficulty swallowing solid and liquid foods, with retching, regurgitation of mucus, and a sensation of “food getting stuck” in his chest. He had constant retrosternal discomfort, appeared malnourished, and complained of halitosis.

## 3. Diagnosis

**Figure 1:**
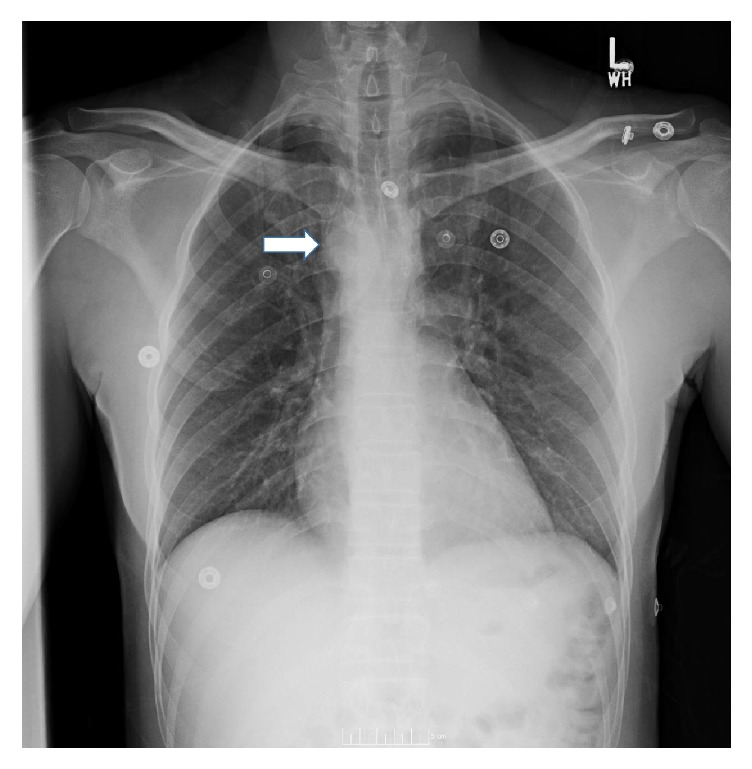
Radiograph of the chest posteroanterior view, with the right sided aortic arch (white arrow).

**Figure 2:**
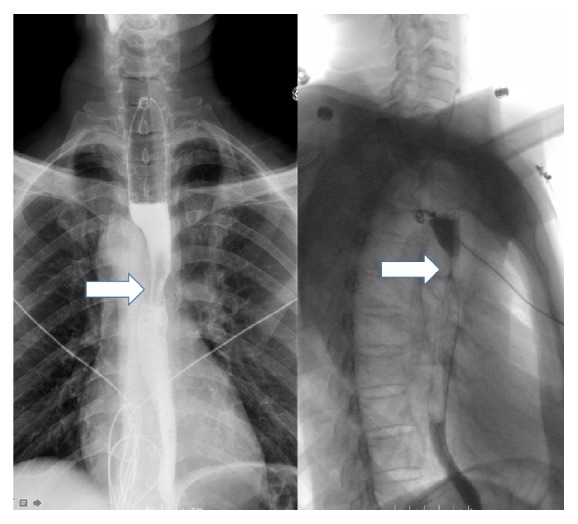
Preoperative barium esophagogram posteroanterior view and lateral view depicting fixed esophageal narrowing at the level of T6 (arrows).

**Figure 3:**
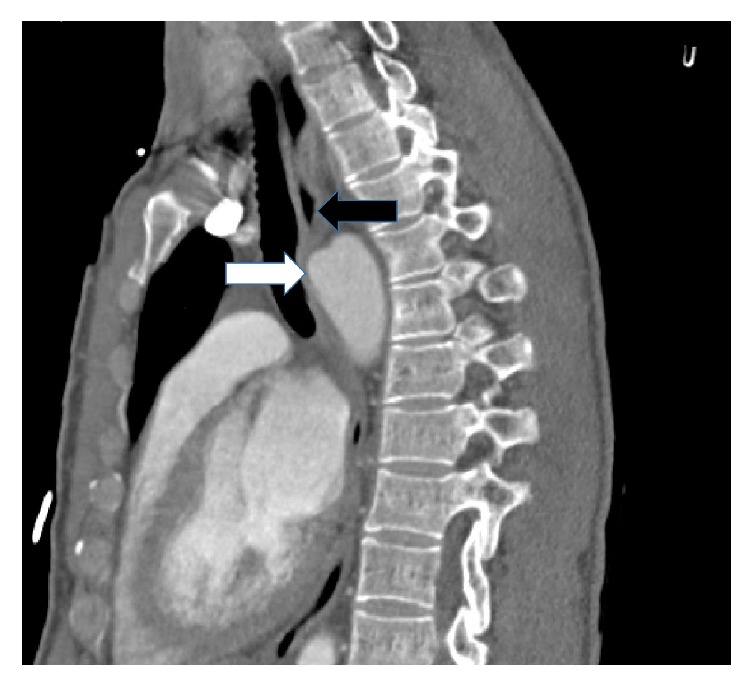
High resolution CT angiogram, sagittal view showing the ductus diverticulum (white arrow) indenting the esophagus (black arrow).

**Figure 4:**
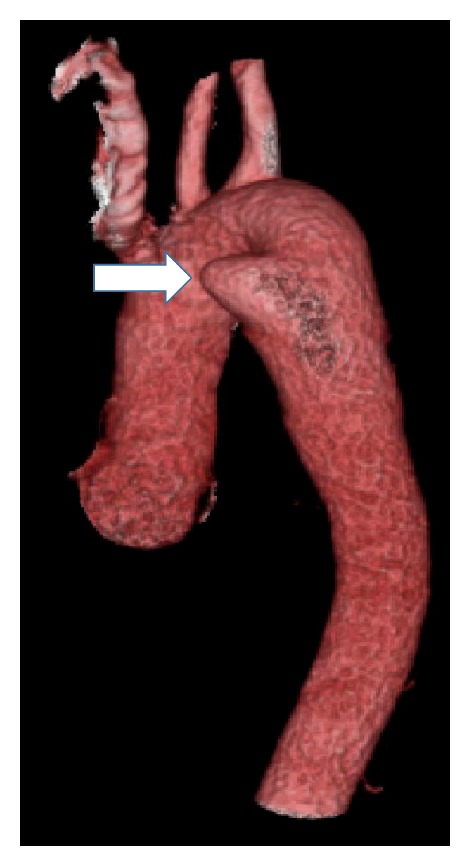
High resolution CT angiogram assisted 3-dimensional reconstruction of the arch of aorta showing the ductus diverticulum (white arrow).

A clear liquid diet was provided during the diagnostic evaluation. The chest radiograph revealed a right sided aortic arch with tracheal deviation to the left (Figure 1). A swallow study confirmed a fixed esophageal narrowing at the level of T6 (Figure 2). Contrast enhanced Computed Tomography (CT) angiogram of the chest and neck revealed a mirror image right aortic arch with a left sided cardiac apex and a prominent ductus diverticulum (measuring 1.7 × 1.8 cm). This structure extended posterior to and indented the mid esophagus (Figure 3). There was no aortic arch dilation with unremarkable pulmonary arteries. High resolution CT angiography with contrast and poststudy 3-dimensional reconstruction is depicted in Figure 4.

## 4. Management

**Figure 5:**
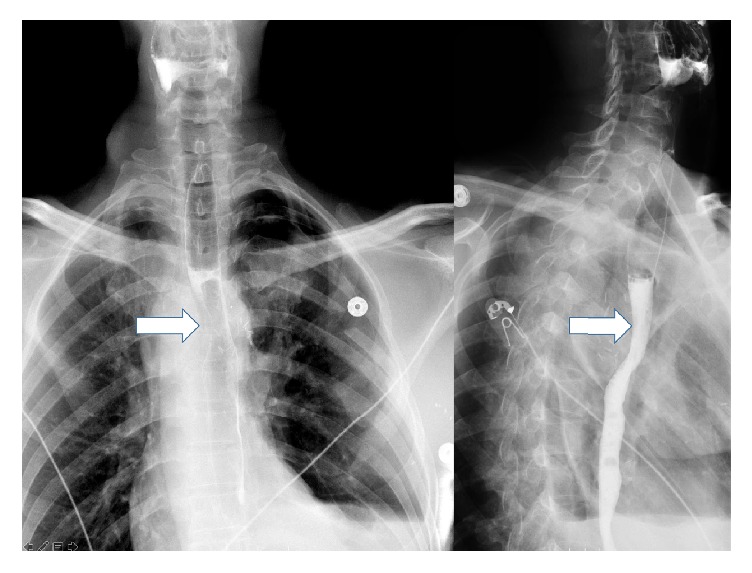
Postoperative barium esophagogram posteroanterior view and lateral view depicting complete resolution of the fixed esophageal narrowing (arrows).

A left posterolateral thoracotomy was performed and the ductus diverticulum was resected. A retroesophageal ligamentum arteriosum was found during surgery and divided. The postoperative course was uneventful, and complete clinical and radiological resolution of the constriction ensued (Figure 5). The patient tolerated food and drink ad lib. He remains asymptomatic.

## 5. Discussion

Right sided aortic arch is a congenital anomaly, which can be a component of a double aortic arch or may be the sole outlet at the level of the aorta. A right sided aortic arch develops from the right 4th branchial instead of the left [1]. This patient's aortic arch exhibited mirror image branching [2] where the arch passed over the right main stem bronchus and continued on as the descending aorta. The branches arising were (1) the left innominate artery, (2) the right carotid artery, and (3) the right subclavian artery.

A ductus diverticulum is a remnant of the infundibular part of the ductus arteriosus. This is a common finding at infancy, present in about 33% of infants, but it involutes over time [3]. A prominent ductus diverticulum is rare and is difficult to visualize on the posteroanterior chest radiograph. On the lateral image it can be seen as a soft tissue mass in the aortopulmonary window. Angiography of the out-pouching is diagnostic, where it is seen as a focal bulge [4]. High resolution CT angiography with contrast and poststudy 3-dimensional reconstruction of the anatomy helped us reach the diagnosis in our case. Multidetector Computed Tomography (MDCT) has been described to differentiate a ductus diverticulum from aneurysms [5].

The simultaneous presence of the aforementioned structures did not explain the extent of esophageal obstruction. The possibility of a vascular ring was thus entertained. A vascular ring refers to a variety of congenital anomalies of the aortic system that can compress the esophagus or trachea causing symptoms [6]. The vascular ring was comprised of the 3 structures externally occluding the esophagus. The first structure was the anteriorly ascending right sided aortic arch; the second structure was the ductus diverticulum laterally. We hypothesized that the third (posterior) component was a retroesophageal ligamentum arteriosum, which had not been identified by preoperative imaging studies. The ligamentum arteriosum can cause significant tracheoesophageal constriction, and its retroesophageal position may cause severe dysphagia [7]. This hypothesis was confirmed during surgical intervention when the retroesophageal ligamentum was visualized and divided.

## 6. Outcome/Follow-Up

This rare combination of congenital anatomical aberrations led to severe dysphagia in our patient. Successful surgical correction in the form of resection of the ductus diverticulum and division of the retroesophageal ligamentum arteriosum led to complete resolution of our patient's symptoms. He continues to do well on regular follow-up.
